# A population-based survey to assess the association between cannabis and quality of life among colorectal cancer survivors

**DOI:** 10.1186/s12885-020-06887-1

**Published:** 2020-05-03

**Authors:** Susan L. Calcaterra, Andrea N. Burnett-Hartman, J. David Powers, Douglas A. Corley, Carmit M. McMullen, Pamala A. Pawloski, Heather Spencer Feigelson

**Affiliations:** 1 Division of General Internal Medicine, Department of Medicine, University of Colorado School of Medicine, 8th Floor, Academic Office 1 Mailstop B180, 12631 E 17th Ave, Aurora, CO 80045 USA; 2 Institute for Health Research, Kaiser Permanente Colorado, Denver, Colorado USA; 3 Department of Gastroenterology, Kaiser Permanente San Francisco, San Francisco, California, USA; 4 Division of Research, Kaiser Permanente Northern California, Oakland, California, USA; 5 Center for Health Research, Kaiser Permanente, Portland, Oregon, USA; 6 HealthPartners Institute, Bloomington, Minnesota USA

**Keywords:** Cannabis, Colorectal cancer, Quality-of-life, Symptomology, Functional status

## Abstract

**Background:**

As more states legalize cannabis for medical and recreational use, people increasingly use cannabis to treat medical conditions and associated symptoms. The prevalence and utility of cannabis for cancer-related symptoms may be clarified by examining cannabis use among patients with a common cancer diagnosis. We aimed to determine the prevalence of cannabis use among colorectal cancer (CRC) survivors and its associations with quality of life (QoL) and cancer-related symptomatology.

**Methods:**

A cross-sectional survey of patient-reported QoL outcomes and behaviors, including cannabis use, was conducted within the Patient Outcomes To Advance Learning network’s (PORTAL) CRC Cohort. The cohort included a population-based sample of healthcare system members ≥18 years old diagnosed with adenocarcinoma of the colon or rectum from 2010 through 2016. We assessed the association between cannabis use and QoL using the European Organization for Research and Treatment of Cancer QLQ-C30 summary score.

**Results:**

Of the 1784 respondents, 293 (16.4%) reported cannabis use following CRC diagnosis. Current tobacco smokers were more likely to use cannabis compared to former or never tobacco smokers (adjusted odds ratio [aOR] 2.71, 95% confidence interval [CI] 1.56 to 4.70). Greater alcohol use (> 4 drinks per month versus ≤4 drinks per month) was associated with cannabis use (aOR 2.17, 95% CI 1.65 to 2.85). There was an association between cannabis use and cancer stage at diagnosis, with stage 3 or 4 CRC patients more likely to use cannabis than stage 1 or 2 CRC patients (aOR 1.68, 95% CI 1.25 to 2.25). After adjusting for demographics, medical comorbidities, stage and site of CRC diagnosis, and prescription opioid use, people who used cannabis had significantly lower QoL than people who did not use cannabis (difference of − 6.14, 95% CI − 8.07 to − 4.20).

**Conclusion:**

Among CRC survivors, cannabis use was relatively common, associated with more advanced stages of disease, associated with tobacco and alcohol use, and not associated with better QoL. Clinicians should inquire about cannabis use among their patients and provide evidence-based recommendations for cancer-related symptoms.

## Background

In 2016, there were an estimated 1.4 million people living with colorectal cancer (CRC) [1]. Patients report physical and emotional complications associated with cancer treatment including nausea, vomiting, pain, depression, anxiety, and fatigue [2, 3]. As more states legalize cannabis for medical and recreational use, people increasingly use cannabis to treat medical conditions and associated symptomatology. Evidence supports the use of cannabis to treat chemotherapy-related nausea and vomiting, though its effectiveness relative to traditional pharmacologic agents is unclear [4]. The role of cannabis to treat pain [5], reduce cancer-related anorexia [6], and improve quality of life among patients living with cancer remains inconclusive [6, 7]. With legalization of cannabis for medical and recreational use, the number of people using cannabis-containing products is likely to continue to increase. As with any other addictive substance, include alcohol or tobacco, it is important that clinicians inquire about their patients’ use of cannabis and to provide evidence-based recommendations to prevent or reduce adverse health effects related to cannabis use. The prevalence and utility of cannabis use for cancer-related symptoms may be clarified by examining cannabis use among patients with a common cancer diagnosis. Using a standardized and comprehensive patient-centered outcome measurement set for patients with CRC, we assessed the use of cannabis on physical and emotional health measures and quality of life (QoL) outcomes among CRC survivors [8]. We hypothesized that cannabis use would not be associated with better patient-reported functional measures nor better QoL measures.

## Methods

This study was conducted within the Patient Outcomes To Advance Learning (PORTAL) network’s CRC Cohort, a retrospective cohort of healthcare system members ≥18 years old and diagnosed with adenocarcinoma of the colon or rectum from January 1, 2010 through December 31, 2016 [9].

The PORTAL CRC cohort included 13,089 people diagnosed with colorectal cancer from 2010 through 2016. Within the PORTAL CRC cohort, four healthcare systems in Minnesota, Colorado, California and Oregon conducted an online survey during July 2018 to October 2018 to assess patient reported QoL and behavioral factors, including cannabis use. We excluded patients with CRC who died (*n* = 4316) or left the health system prior to June 2018 (*n* = 1282), those without an available email address (*n* = 1005), and those with in situ CRC, prior cancer, or previous colectomy (*n* = 625). We further excluded people who were listed on the “do not contact list” (*n* = 61), who did not successfully receive the survey invitation (*n* = 165), and who did not complete the survey (*n* = 3829). Finally, we excluded people who did not answer survey questions regarding cannabis use (*n* = 22). Our final sample include 1784 participants. The study was approved by the Kaiser Permanente Colorado Institutional Review Board. Written consent was obtained from all participants.

Patients were invited via email to complete a survey that included the European Organization for Research and Treatment of Cancer (EORTC) Quality of Life Questionnaire Core 30 (QLQ-C30) to assess patient-reported measures of functional and symptom-related outcomes and other survey domains [10]. Survey questions were categorized into the following sections: family history of colorectal cancer; lifestyle and habits; medication use; pain and medication use; treatment, side effects and life after cancer; ostomy; demographics. Cannabis use (yes/no) was determined by participant responses to the following questions: “Have you used marijuana after you were diagnoses with colon or rectal cancer? (yes/no)”, “After you were diagnosed with colon or rectal cancer, about how many times have you used marijuana? (1 or 2 times; 3 – 10 times; 11 – 40 times; 41-99 times; 100 or more times)”, “Have you used marijuana in the last 30 days? (yes/no)”, “During the last 30 days, on how many days did you use marijuana? (write in response)”, “During the last 30 days, did you used marijuana for medical or health reasons, recreational reasons only, both medical and recreational reasons?” Participants were dichotomized into two groups, those who did use cannabis use following their CRC diagnosis and those who did not use cannabis following their CRC diagnosis, based upon their survey responses. Potency or route of cannabis use was not captured within the survey questions. Quantification of cannabis use was not included in data analysis. Demographic and medical data were extracted from health records.

### Statistical analyses

We used logistic regression to estimate unadjusted and adjusted odds ratios (aORs) and 95% confidence intervals (CIs) to compare the odds of cannabis use after CRC diagnosis to patient demographics, health behaviors, comorbidity burden [11], and tumor characteristics.

For QoL measures, we calculated functional and symptom scales ranging from 0 to 100 [10]. Functional scales included: physical, social, role, emotional, and cognitive domains. For functional scales, a higher score represented a higher or healthier level of functioning. Symptom scales included: fatigue, nausea, vomiting, pain, dyspnea, insomnia, appetite loss, constipation, and diarrhea. For symptoms scales, a higher score indicated a higher level of symptomatology or worse symptoms. We calculated a QLQ-C30 summary score where higher scores indicated better functioning and fewer symptoms [12]. We used linear regression models to estimate unadjusted and adjusted mean differences and 95% confidence intervals (CI) for each score comparing people with CRC who used cannabis to people with CRC who did not use cannabis. Table 2 lists the covariates in the adjusted model. We conducted sensitivity analyses, stratified by time from CRC diagnosis to survey completion (≤ 2 years vs. > 2 years).

### Power calculation

Using PASS 15 Power Analysis and Sample Size Software (2017) (NCSS, LLC. Kaysville, Utah), we calculated that given our sample size and α = 0.05, our study had 80% power to detect a mean difference in overall QoL score of 3 or greater.

## Results

We distributed 5635 surveys and 1784 (31.6%) CRC survivors, between 1.48 and 8.62 years from the time of their diagnosis, responded. Overall, 293 (16.4%) patients reported cannabis use following their CRC diagnosis; among these 293, 93 (31.7%) used cannabis ≥100 times, 67 (22.9%) used cannabis 3 to 10 times, and 163 (55.6%) used cannabis during the 30 days prior to survey completion.

Cannabis use was more common among younger survivors, tobacco smokers, and those with greater alcohol use (Table 1). Cannabis use was more likely in patients diagnosed with stage 3 or 4 CRC than stage 1 or 2 CRC (adjusted odds ratio [aOR] 1.68, 95% CI 1.25 to 2.25).

**Table 1:** Demographic Data and Self-Reported Conditions of Colorectal Cancer (CRC) Survivors

	**No Cannabis Use after CRC Diagnosis**	**Cannabis Use after CRC Diagnosis**	**Unadjusted OR (95% CI)**	**Adjusted OR**^**a**^ **(95% CI)**
	**N (%)**	**N (%)**		
**Overall**	1491 (83.6%)	293 (16.4%)		
**Age (years)**				
18–49	91 (6.1%)	41 (14.0%)	Ref	
50–64	451 (30.2%)	135 (46.1%)	0.664 (0.44, 1.01)	0.66 (0.42, 1.03)
65+	949 (63.6%)	117 (39.9%)	0.27 (0.18, 0.42)	0.28 (0.18, 0.44)
**Race/ethnicity**				
Non-Hispanic White	1214 (81.4%)	253 (86.3%)	Ref	
Non-Hispanic Black	55 (3.7%)	12 (4.1%)	1.05 (0.55, 1.98)	1.06 (0.53, 2.12)
Asian/Pacific Islander	134 (9.0%)	12 (4.1%)	0.43 (0.23, 0.79)	0.36 (0.19, 0.67)
Hispanic	88 (5.9%)	16 (5.5%)	0.872 (0.50, 1.51)	0.75 (0.42, 1.35)
**Gender**				
Female	766 (51.4%)	131 (44.7%)	Ref	
Male	725 (48.6%)	162 (55.3%)	1.31 (1.02, 1.68)	1.31 (1.00, 1.72)
**Education**				
No college	245 (16.4%)	38 (13.0%)	Ref	
Some college or College degree	1246 (83.6%)	255 (87.0%)	1.32 (0.91, 1.91)	1.16 (0.79, 1.72)
**Marital Status**				
Not married /partnered	471 (31.6%)	98 (33.4%)	Ref	
Married or partnered, living as married	1020 (68.4%)	195 (66.6%)	0.92 (0.70, 1.20)	0.75 (0.56, 1.00)
**Healthcare system**^b^				
A	1141 (76.5%)	231 (78.8%)	Ref	
B	155 (10.4%)	33 (11.3%)	1.05 (0.70, 1.57)	1.02 (0.66, 1.57)
C	160 (10.7%)	22 (7.5%)	0.68 (0.43, 1.09)	0.75 (0.46, 1.23)
D	35 (2.3%)	7 (2.4%)	0.99 (0.43, 2.25)	0.78 (0.33, 1.85)
**Smoking status**				
Never or Former	1445 (96.9%)	269 (91.8%)	Ref	
Current	46 (3.1%)	24 (8.2%)	2.80 (1.68, 4.67)	2.71 (1.56, 4.70)
**Alcohol use**				
≤ 4 times per month	1032 (69.2%)	146 (49.8%)	Ref	
> 4 times per month	459 (30.8%)	147 (50.2%)	2.26 (1.76, 2.92)	2.17 (1.65, 2.85)
**Charlson comorbidity score**				
0	462 (31.0%)	110 (37.5%)	Ref	
1 or 2	880 (59.0%)	168 (57.3%)	0.80 (0.62, 1.05)	1.07 (0.80, 1.44)
3+	149 (10.0%)	15 (5.1%)	0.42 (0.24, 0.75)	0.741 (0.40, 1.36)
**Stage**				
1 or 2	842 (56.5%)	120 (41.0%)	Ref	
3 or 4	453 (30.4%)	124 (42.3%)	1.92 (1.46, 2.53)	1.68 (1.25, 2.25)
Unstaged/Unknown	196 (13.1%)	49 (16.7%)	1.75 (1.22, 2.53)	1.52 (1.02, 2.26)
**Anatomical Site**				
Colon	1076 (72.2%)	176 (60.1%)	Ref	
Rectum	415 (27.8%)	117 (39.9%)	1.72 (1.33, 2.24)	1.30 (0.98, 1.73)

^a^ Adjusted for all other variables in the table

^b^ Specific site names are masked for confidentiality

After adjusting for demographics, comorbidities, stage and site of cancer diagnosis, and prescription opioid use, the mean summary QoL score was 6.14 points lower (95% CI − 8.07 to − 4.20) in people who used cannabis compared to people who did not use cannabis. People who used cannabis reported lower functioning roles (all statistically significant) and higher symptom scores (all statistically significant except for diarrhea) compared to people who did not use cannabis (Table 2).

**Table 2:** Association of Cannabis Use on Quality of Life Summary Score and other Self-Reported Outcomes

	**Cannabis use (Yes) (*n*** **= 293)**	**Cannabis use (No) (*n*** **= 1491)**	**Unadjusted (95%CI)**	**Adjusted**^**a**^**(95% CI)**
	**Mean Score**			
**QLQ-C30 Summary Score**^b^	**76.27**	**83.07**	**−6.80 (−8.79, −4.80)**	**− 6.14 (− 8.07, − 4.20)**
Physical functioning^c^	86.13	85.07	1.05 (−1.36, 3.47)	−2.75 (−5.01, −0.49)
Social functioning^c^	69.85	81.52	−11.67 (− 15.06, − 8.28)	−9.84 (− 13.22, − 6.46)
Role functioning^c^	73.29	82.01	− 8.72 (−12.23, − 5.22)	− 7.54 (− 11.03, − 4.04)
Emotional functioning^c^	71.59	81.71	−10.11 (− 12.70, − 7.52)	−7.74 (− 10.35, − 5.12)
Cognitive functioning^c^	74.12	83.11	−8.99 (− 11.61, − 6.37)	−8.60 (− 11.29, − 5.90)
Fatigue^d^	32.92	24.73	8.18 (5.13, 11.24)	8.26 (5.23, 11.29)
Nausea & vomiting^d^	9.70	4.85	4.85 (3.11, 6.59)	3.29 (1.52, 5.07)
Pain^d^	24.74	15.29	9.45 (6.31, 12.60)	7.58 (4.52, 10.63)
Dyspnea^d^	17.41	13.76	3.65 (0.69, 6.61)	4.53 (1.53, 7.53)
Insomnia^d^	34.70	26.65	8.05 (4.46, 11.64)	7.13 (3.43, 10.82)
Appetite loss^d^	17.13	8.82	8.30 (5.70, 10.90)	7.21 (4.55, 9.87)
Constipation^d^	22.49	17.76	4.73 (1.49, 7.97)	4.38 (1.04, 7.73)
Diarrhea^d^	24.63	22.73	1.90 (−1.58, 5.38)	0.83 (−2.77, 4.43)

^a^ Adjusted for age, race/ethnicity, gender, education, marital status, health plan, smoking, alcohol use, Charlson comorbidity index [11], stage, site, opioid use after diagnosis

^b^ A high score for QoL summary score represents a high QoL

^c^ A high score for a functional scale represents a high / healthy level of functioning

^d^ A high score for a symptom scale / item represents a high level of symptomatology / problems

In a sensitivity analysis of recency of CRC diagnosis (> 2 years; ≤ 2 years), there were no statistically significant differences in the results by recency of CRC diagnosis. However, all estimates within ≤2 years of diagnoses were more extreme than estimates within > 2 years of diagnosis, indicating worse functioning and symptom severity for the cannabis use group (data not shown).

## Discussion

We surveyed over 1700 CRC survivors and found cannabis use was not associated with improved QoL or cancer-related function or symptoms compared to CRC survivors without cannabis use. Previous studies report conflicting evidence regarding the use of cannabis to improve QoL among oncology patients [6, 7]. Our results suggest that, although use is relatively common among patients with CRC, they do not have improved QoL compared to non-users, even after adjusting for baseline factors. Our findings may have differed because we limited our sample to CRC patients, potential confounding by indication (whereby more symptomatic patients were users), and because the tool used to measure outcomes in this study was developed to assess patient-centered outcomes among CRC patients [8].

Despite the observation that cannabis use was associated with lower QoL, functional status, and symptomatology, approximately 16% of patients in this study reported cannabis use, with the majority of recent users reporting medically-indicated use. As states increasingly legalize cannabis, more patients will likely use cannabis to treat cancer-related symptoms. Current evidence suggests that clinicians may lack the knowledge to properly advise patients about cannabis use, including in the context of co-use with alcohol and tobacco. In a survey of 400 oncology providers practicing across the United States, 70% reported insufficient knowledge to make clinical recommendations about cannabis use in people living with cancer [13]. A survey of 494 health care providers in Washington state found that 64.4% had limited knowledge of cannabis-containing products or where to get them [14]. Conversely, a survey of adult patients with a cancer diagnosis from a state with legalized cannabis found that 75% of respondents would like to have received education about cannabis use from their medical team, but only 15% received information [15]. Our results demonstrated that patients who used cannabis generally reported feeling worse than patients who did not use cannabis. Whether patients who used cannabis discussed their cannabis use with their medical team is unknown.

Our study has several strengths, including a well-defined population of CRC survivors and the use of validated, CRC specific QoL measures [10, 12]. Our study limitations should also be acknowledged. Patients were excluded from this study if they lacked an email address, did not successfully receive the survey or did not complete the survey, it they died or departed from the CRC cohort, and if they did not want to be contacted. Thus, it is possible we introduced attrition bias, susceptibility bias, or self-selection bias into our study results because the final study population was not representative of the entire CRC cohort. Next, the cross-sectional design does not allow us to directly evaluate effectiveness or to examine cannabis use prior to CRC diagnosis. Most of the patients surveyed would have completed treatment several years prior to receiving the survey. Interestingly, results from our sensitivity analysis found lower QoL scores among people who used cannabis and completed the survey within 2 years of their CRC diagnosis. This finding suggests increased symptomatology related to recency of diagnosis, though these results were not statistically significant. Patients with more severe symptoms and reduced function may be more likely to use cannabis, resulting in confounding by indication. While states are increasingly legalizing medical and recreational cannabis, it remains a United States Drug Enforcement Administration (DEA) schedule I substance. For this reason, patients may be reluctant to report cannabis use on the survey, leading to misclassification and attenuation of our results. Finally, the effect of cannabis on cancer-related symptoms may correlate with the potency of the tetrahydrocannabinol (THC), its ratio of THC to cannabidiol (CBD) in the cannabis product, the frequency of use (daily, weekly, monthly) and route of use (edibles, smoked). In our study, there was a wide range of frequency of cannabis use and data on THC potency or ratio was not collected in the survey thus was unavailable.

## Conclusion

Cannabis use was associated with lower QoL and functional status among CRC survivors. Despite inconclusive evidence to support cannabis use to manage cancer-related symptomatology, patients will likely continue to use cannabis. Health care providers should inquire about cannabis use among their patients with cancer. They should provide their patients with current evidence-based recommendations for cancer-related symptoms and explore their patient’s goals for cannabis use. In this way, clinician may better understand how to best help their patients improve their functional status, reduce their symptom burden, and maximize their patient’s quality of life.

## Data Availability

Not applicable.

## Abbreviations

QOL: Quality of life
CRC: Colorectal cancer
aOR: Adjusted odds ratio
CI: Confidence interval
EORTC: European Organization for Research and Treatment of Cancer
QLQ-C30: Quality of Life Questionnaire Core 30
THC: Tetrahydrocannabinol
CBD: Cannabidiol

## References

[1] Miller KD, Siegel RL, Lin CC (2016). Cancer treatment and survivorship statistics, 2016. CA Cancer J Clin.

[2] Hofman M, Morrow GR, Roscoe JA (2004). Cancer patients' expectations of experiencing treatment-related side effects: a University of Rochester Cancer Center--Community Clinical Oncology Program study of 938 patients from community practices. Cancer..

[3] van den Beuken-van Everdingen MH, Hochstenbach LM, Joosten EA, Tjan-Heijnen VC, Janssen DJ. Update on Prevalence of Pain in Patients With Cancer: Systematic Review and Meta-Analysis. J Pain Symptom Manage. 2016;51(6):1070–90 e1079. 10.1016/j.jpainsymman.2015.12.340 27112310

[4] Smith LA, Azariah F, Lavender VT, Stoner NS, Bettiol S. Cannabinoids for nausea and vomiting in adults with cancer receiving chemotherapy. Cochrane Database Syst Rev. 2015;11. 10.1002/14651858.CD009464.pub2 PMC6931414 26561338

[5] Mucke M, Phillips T, Radbruch L, Petzke F, Hauser W. Cannabis-based medicines for chronic neuropathic pain in adults. Cochrane Database Syst Rev. 2018;3:Cd012182. 10.1002/14651858.CD012182.pub2 PMC6494210 29513392

[6] Strasser F, Luftner D, Possinger K (2006). Comparison of orally administered Cannabis extract and Delta-9-Tetrahydrocannabinol in treating patients with Cancer-related anorexia-cachexia syndrome: a multicenter, phase III, randomized, double-blind, placebo-controlled clinical trial from the Cannabis-in-cachexia-study-group. J Clin Oncol.

[7] Zhang H, Xie M, Archibald SD, Jackson BS, Gupta MK (2018). Association of Marijuana use with Psychosocial and Quality of life outcomes among patients with head and neck Cancer. JAMA Otolaryngol Head Neck Surg.

[8] Zerillo JA, Schouwenburg MG, van Bommel ACM (2017). An international collaborative standardizing a comprehensive patient-centered outcomes measurement set for colorectal Cancer. JAMA Oncol.

[9] Feigelson HS, McMullen CK, Madrid S (2017). Optimizing patient-reported outcome and risk factor reporting from cancer survivors: a randomized trial of four different survey methods among colorectal cancer survivors. J Cancer Surviv.

[10] Aaronson NK, Ahmedzai S, Bergman B (1993). The European Organization for Research and Treatment of Cancer QLQ-C30: a quality-of-life instrument for use in international clinical trials in oncology. J Natl Cancer Inst.

[11] Charlson ME, Pompei P, Ales KL, MacKenzie CR (1987). A new method of classifying prognostic comorbidity in longitudinal studies: development and validation. J Chronic Dis.

[12] Giesinger JM, Kieffer JM, Fayers PM (2016). Replication and validation of higher order models demonstrated that a summary score for the EORTC QLQ-C30 is robust. J Clin Epidemiol.

[13] Braun IM, Wright A, Peteet J (2018). Medical oncologists’ beliefs, practices, and knowledge regarding marijuana used therapeutically: a nationally representative survey study. J Clin Oncol.

[14] Carlini BH, Garrett SB, Carter GT (2017). Medicinal Cannabis: a survey among health care providers in Washington state. Am J Hosp Palliative Care.

[15] Pergam SA, Woodfield MC, Lee CM (2017). Cannabis use among patients at a comprehensive cancer center in a state with legalized medicinal and recreational use. Cancer..

